# Unique Contribution of Haptoglobin and Haptoglobin Genotype in Aneurysmal Subarachnoid Hemorrhage

**DOI:** 10.3389/fphys.2018.00592

**Published:** 2018-05-31

**Authors:** Spiros L. Blackburn, Peeyush T. Kumar, Devin McBride, Hussein A. Zeineddine, Jenna Leclerc, H. Alex Choi, Pramod K. Dash, James Grotta, Jaroslaw Aronowski, Jessica C. Cardenas, Sylvain Doré

**Affiliations:** ^1^Department of Neurosurgery, The University of Texas Houston Health Sciences Center, Houston, TX, United States; ^2^Department of Anesthesiology, University of Florida, College of Medicine, Gainesville, FL, United States; ^3^Department of Neurology, The University of Texas Health Sciences Center, Houston, TX, United States; ^4^Department of Surgery, Division of Acute Care Surgery and Center for Translational Injury Research, The University of Texas Health Science Center, Houston, TX, United States; ^5^Departments of Neurology, Psychiatry, Psychology, Pharmaceutics, and Neuroscience, University of Florida, McKnight Brain Institute, Gainesville, FL, United States

**Keywords:** cerebral vasospasm, genetic biomarker, heme, microthrombosis, neuroinflammation, personalized medicine, prognostic marker, subarachnoid hemorrhage

## Abstract

Survivors of cerebral aneurysm rupture are at risk for significant morbidity and neurological deficits. Much of this is related to the effects of blood in the subarachnoid space which induces an inflammatory cascade with numerous downstream consequences. Recent clinical trials have not been able to reduce the toxic effects of free hemoglobin or improve clinical outcome. One reason for this may be the inability to identify patients at high risk for neurologic decline. Recently, haptoglobin genotype has been identified as a pertinent factor in diabetes, sickle cell, and cardiovascular disease, with the Hp 2-2 genotype contributing to increased complications. Haptoglobin is a protein synthesized by the liver that binds free hemoglobin following red blood cell lysis, and in doing so, prevents hemoglobin induced toxicity and facilitates clearance. Clinical studies in patients with subarachnoid hemorrhage indicate that Hp 2-2 patients may be a high-risk group for hemorrhage related complications and poor outcome. We review the relevance of haptoglobin in subarachnoid hemorrhage and discuss the effects of genotype and expression levels on the known mechanisms of early brain injury (EBI) and cerebral ischemia after aneurysm rupture. A better understanding of haptoglobin and its role in preventing hemoglobin related toxicity should lead to novel therapeutic avenues.

## Introduction

Aneurysmal subarachnoid hemorrhage (aSAH) affects more than 30,000 people per year in the United States, with an estimated 30–50% mortality (Hop et al., 1997; Stegmayr et al., 2004; Zacharia et al., 2010). It is the deadliest form of stroke. Although the initial hemorrhage and early brain injury (EBI) is a major cause of poor outcomes, delayed mechanisms of brain injury also play a significant role.

Cerebral vasospasm (CV) and delayed cerebral infarcts (DCI) are the most studied delayed events and are known to contribute to poor outcomes after aSAH (Rosengart et al., 2007). Nevertheless, extensive basic research and clinical trials targeting CV have not delivered significant progress likely due to the fact that cerebral ischemia also occurs in regions without clear angiographic vasospasm (Gomis et al., 2010; Macdonald et al., 2011). Furthermore, CV is only one mechanism contributing to poor outcomes in addition to inflammation, cerebral microthrombosis, blood brain barrier (BBB) dysfunction and cortical spreading depolarizations (Neil-Dwyer et al., 1994; Stein et al., 2006; Vergouwen et al., 2008; Dreier et al., 2009; Dhar et al., 2012; Woitzik et al., 2012). As the cerebral injury is largely multifactorial, targeting one aspect may have limited efficacy in reducing delayed brain injury.

Following aneurysmal rupture, a transient increase in intracranial pressure and a decrease in cerebral blood flow result in the initiation of an EBI cascade that includes global cerebral ischemia and cerebral edema (Bederson et al., 1995; Claassen et al., 2002; Schubert et al., 2011). Much of the evidence for this comes from animal studies rather than clinical studies due to the inability to monitor the ictus, and is reviewed elsewhere (Sehba et al., 2012; Sabri et al., 2013b). In animal models, this cascade results in inflammation, endothelial cell necrosis/apoptosis, neuronal apoptosis, and autoregulatory dysfunction and remains a critical area of active research.

With this EBI, blood is released into the subarachnoid space, mixing with cerebrospinal fluid (CSF), and forming a clot. Within 24 h following aSAH, an intense polymorphonuclear leukocyte infiltration of the meninges is seen (Macdonald and Weir, 1991). Phagocytosis (targeting hematoma clearance) and lysis of red blood cells (RBCs) occurs by 16–32 h, but continues for days, with clumps of intact RBCs still enmeshed in the arachnoid for up to 35 days after aSAH (Macdonald and Weir, 1991; Foreman, 2016). It is estimated that >250 million molecules of hemoglobin (Hb) are released per single RBC (Wintrobe and Greer, 2004). The inert free Hb, unless bound by its high affinity binding protein haptoglobin (Hp) will quickly undergo oxidation and become a potent pro-inflammatory and cytotoxic molecule (Foley et al., 1994; Ascenzi et al., 2005). In binding with Hb, Hp prevents oxidation and thus confers a neuroprotective role (Zhao et al., 2009; Andersen et al., 2012).

In humans, there are two alleles for Hp, and a number of known rare variants (Langlois and Delanghe, 1996). The role of Hp genotype and its relevance to disease severity has been studied in different specialties including epilepsy, cardiovascular disease, diabetes, sickle cell, and renal disease (Panter et al., 1985; Langlois and Delanghe, 1996; Asleh et al., 2003; Sadrzadeh et al., 2004; Amiri et al., 2013; MacKellar and Vigerust, 2016). The overall conclusion of these studies is that the Hp2-2 genotype is associated with a more severe disease phenotype, and with increased immune reactivity (Langlois and Delanghe, 1996; Roguin et al., 2003; Levy et al., 2007, 2010; Goldenstein et al., 2012; Purushothaman et al., 2012).

Three previous studies have demonstrated a link between Hp genotype and incidence of CV following aSAH (Borsody et al., 2006; Ohnishi et al., 2013; Leclerc et al., 2015). In a fourth study (the largest study with 193 patients), Kantor et al. identified Hp2-2 as a predictor of poor outcomes but did not evaluate DCI or cerebral vasospasm (Kantor et al., 2014). The association between poor outcome and Hp 2-2 has been reproduced in one study, though contested in another (Ohnishi et al., 2013; Leclerc et al., 2015). Taken as a whole, this data implicates Hp genotype has the potential to be an important mediator in long term patient outcome after aSAH. In this review, we discuss the role that Hp and Hp polymorphism plays, and how this influences the mechanisms of delayed injury following aSAH along with supporting evidence. Understanding the role of Hp on the clearance of hemoglobin from the CSF and brain, and its mitigation of pathological mediators after aSAH may lead to novel therapeutics.

## Extracellular hemoglobin processing and the role of haptoglobin

**Figure 1:**
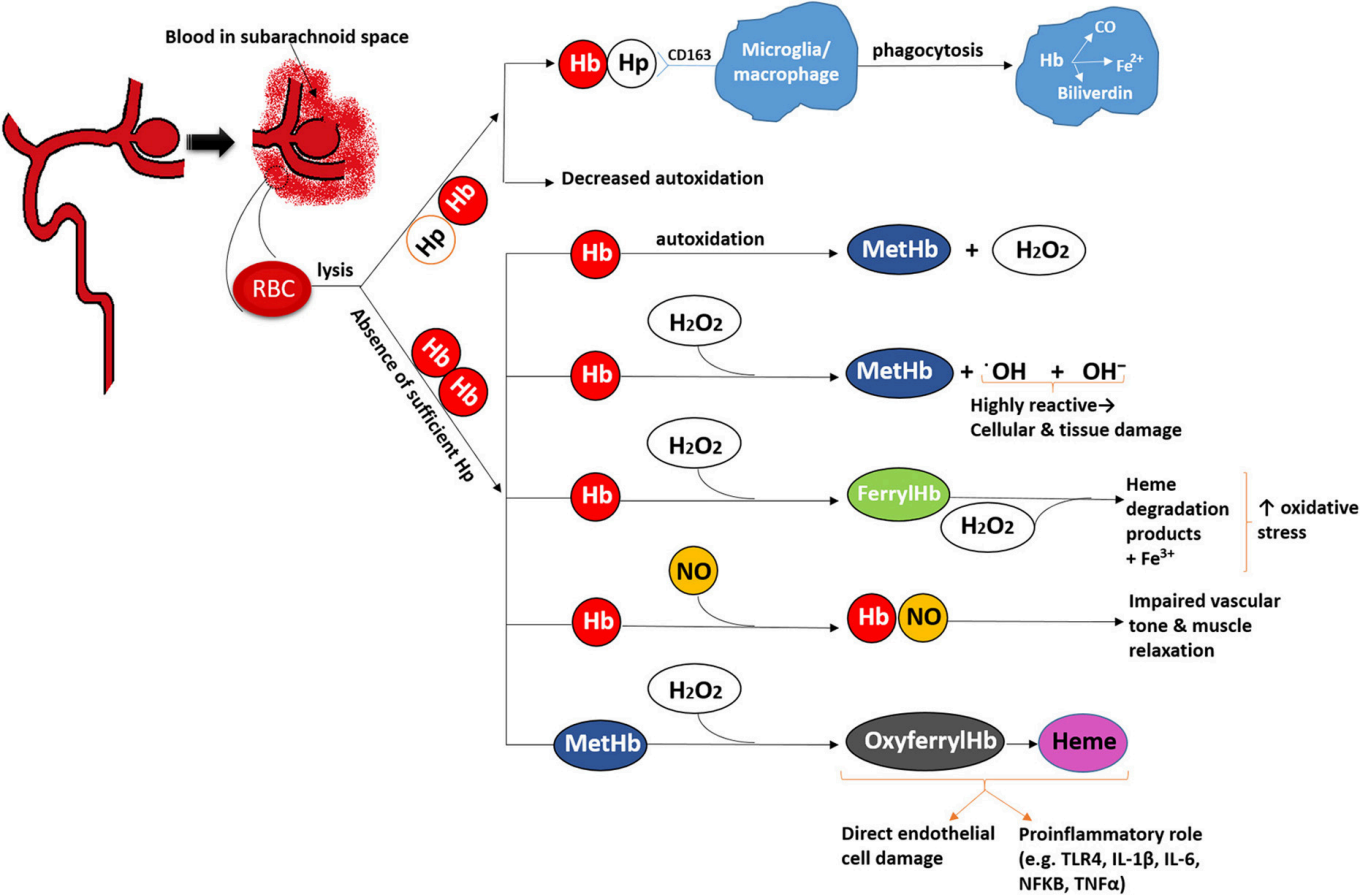
Overall representation of the role of haptoglobin.

Under normal conditions, Hb is confined to the RBC, and intracellular Hb undergoes auto-oxidation at a slow rate, producing methemoglobin (metHb) and reactive oxygen species (ROS), primarily superoxide anion (Shikama, 1998; Boutaud et al., 2010). However, an extensive antioxidant system within the RBCs is responsible for reducing the oxidized Hb and neutralizing the ROS (Gonzales et al., 1984; Lee et al., 2003; Nagababu et al., 2003) (Figure 1).

Extracellular Hb (i.e., after RBC lysis) will undergo a series of oxidative reactions generating metHb, which undergoes further oxidation producing free heme and free iron (Rifkind et al., 2014). Considering that 250–270 million molecules of Hb are released per RBC, and that each Hb contains four hemes (also called iron-protoporphyrin IX), RBC lysis quickly creates levels of Hb that the brain cells cannot efficiently metabolize. These Hb molecules are the source of pathological reactions including production of free radicals, induction of proinflammatory cascades, endothelial and smooth muscle cell dysfunction and injury, and reduced NO availability (Sadrzadeh et al., 1984; Touyz and Schiffrin, 2004; Figueiredo et al., 2007; Azarov et al., 2008; Silva et al., 2009; Cabrales and Friedman, 2013; Lisk et al., 2013). Iron imparts significant cell damage and neuronal death following aSAH, though some argue that the iron binding protein ferritin could be sufficient to mitigate iron-toxicity. Deleterious effects of iron were suggested to be reduced in rat SAH models treated with the iron chelator, deferoxamine (Lee et al., 2010). However, toxicity related to clinical use of deferoxamine would limit its use.

Haptoglobin is an acute phase reactant primarily produced by the liver and is the major Hb binding plasma protein. Formation of Hp-Hb complex mediates clearance of Hb by internalization of this complex through scavenger membrane receptor CD163 on monocytes and macrophages in the liver and spleen, and potentially microglia within the brain in case of cerebral bleeding (Schaer C. A. et al., 2013; Schaer D. J. et al., 2013; Rifkind et al., 2014; Schaer et al., 2014). Once internalized, the heme is degraded by heme oxygenase 1 (HO1) into biliverdin and iron (Thomsen et al., 2013). The HO1 is induced by heme as well as inflammatory cytokines (Terry et al., 1998; Thomsen et al., 2013).

In the absence of sufficient Hp reserve (e.g., hypohaptoglobinemia), Hb structure is often modified by the oxidation which reduces ability of CD163 to bind the complex (Vallelian et al., 2008). Hp-Hb binding stabilizes the Hb structure, prevents Hb protein oxidation, and preserves CD163 binding such that the Hb can be effectively endocytosed by the phagocyte, and then metabolized by heme oxygenase (Buehler et al., 2009). The protective mechanisms of Hp are particularly relevant in the intravascular space where hemolysis often occur. However, Hp although present in the CSF, is not as abundant as in the blood and is readily overwhelmed by the molar ratio of Hb after SAH (Galea et al., 2012). Presumably, this facilitates heme-iron induced oxidative stress and an exuberant pro-inflammatory cascade with consequences including vascular injury, cerebral vasospasm, microthrombosis, and neuronal toxicity. In intracerebral hemorrhage (ICH) models, experimentally-induced hypohaptoglobimemia resulted in increased oxidative stress in the peri-hematoma tissue, while animals overexpressing human Hp demonstrated less neurological damage and less injury to the neurons and oligodendroglia within the ICH-affected brain (Zhao et al., 2009).

Two major alleles, Hp1 and Hp2, exist for the Hp gene found on chromosome 16. The two alleles are responsible for three different possible genotypes with structural polymorphism: homozygous (1-1 or 2-2) and heterozygous 2-1. In Western populations, it is estimated that the distribution of Hp 1-1 is ~16%, Hp 2-1 is ~48%, and Hp 2-2 is ~36%. Hp is cleaved into two subunits α and β chains, joined by a disulfide bond. Both alleles share the same β chain (Goldenstein et al., 2012). The β chain is responsible for binding the Hb, thus both genotypes have similar Hb binding affinity (Asleh et al., 2005). However, the Hp-Hb complex formed with Hp 1-1 is a superior antioxidant compared to Hp 2-2, and Hp 1-1 patients have higher circulating Hp levels (Langlois and Delanghe, 1996; Melamed-Frank et al., 2001; Dalan et al., 2016, 2017). In addition, the circulating Hp1-1 bound hemoglobin is cleared faster than its Hp 2 counterpart (Asleh et al., 2003; Azarov et al., 2008).

## Neuroinflammation

Neuroinflammation ensues following aSAH with increased chemokines and cytokines in CSF and serum. Certain cytokines, specifically IL-1β, TNFα, and IL-6, have been repeatedly linked to worse clinical outcome, increased CV and worsened neurological decline in patients with aSAH (Mathiesen et al., 1997; Gruber et al., 2000; Kwon and Jeon, 2001; Hendryk et al., 2004; Chou et al., 2012). Investigation into the CSF of patients with aSAH has shown an increase in the number of activated neutrophils and monocytes, and that the robustness in this response correlates with delayed neurologic deterioration (Provencio et al., 2010; Moraes et al., 2015). Murine studies depleting peripheral neutrophils prior to the injury improved cognitive outcomes after aSAH (Provencio et al., 2016). Fever, increased white blood count, heart rate or tachypnea is seen in up to 87% of patients, and this inflammatory response increases the risk of developing delayed cerebral ischemia (Dhar and Diringer, 2008).

The critical roles played by Toll-like receptors (TLR) in SAH inflammation have been an area of active research in recent years. TLRs belong to the pattern recognition receptors family and play a well-described role in innate immunity and inflammatory responses (Lee and Lee, 2002). TLR4 is the one most studied in neuroinflammation and SAH. It is expressed in different cell types including microglia, neurons, astrocytes and endothelial cells. TLR4 (and other family members such as TLR2) can be activated by a number of ligands including heme and other components released by SAH (Lee and Lee, 2002; Jack et al., 2005).

Recently, in addition to heme, metHb was established as a ligand for TLR4 (Figueiredo et al., 2007; Kwon et al., 2015). In aSAH, TLR4 binding metHb/heme would result in the activation of a nuclear factor kappa beta (NFκB)-mediated pro-inflammatory cascade within microglia (or macrophages), DNA transcription, and production and release of TNFα and IL-1β (Lehnardt, 2010; Kwon et al., 2015; Lucke-Wold et al., 2016). Haptoglobin is relevant to this TLR4 inflammatory pathway since Hp-Hb binding prevents conversion of the oxyHb to metHb, as well as extracellular release of heme from hemoglobin (Schaer et al., 2014). Other forms of hemoglobin, such as oxyHb, do not bind TLR4 (Gram et al., 2013; Schaer et al., 2014; Kwon et al., 2015). The TLR4 pathway in microglia results in the release of chemokines/cytokines and recruitment of activated peripheral macrophages and neutrophils into the CNS (Olson and Miller, 2004). Animal studies have shown that such microglial activation results in neuronal cell injury, and conversely depletion of microglia is neuroprotective (Hanafy, 2013; Schneider et al., 2015).

Macrophages also play a central role in regulating senescent RBCs and Hb clearance (Soares and Hamza, 2016). Although under maintenance conditions the erythrophagocyte acts in an anti-inflammatory capacity, excess free heme and iron can modulate macrophage phenotype into a proinflammatory M1 phenotype producing inflammatory cytokines and ROS (Soares and Hamza, 2016; Vinchi et al., 2016). This free heme/iron induced differentiation mechanism depends on TLR4 signaling pathway and ROS (Vinchi et al., 2016). Interestingly, heme scavengers and iron chelators, may mitigate macrophage polarization into the proinflammatory phenotype (Lin et al., 2010; Vinchi et al., 2016).

In disease states including diabetes and obesity, Hp 2-2 has been linked to a higher inflammatory state with elevations in WBC, TNFα, and IL-6 (Lazalde et al., 2014; Costacou et al., 2015). The Hp 2-2 is also over-represented in autoimmune diseases including lupus and rheumatoid arthritis (MacKellar and Vigerust, 2016). In inflammatory states without CSF blood products, TNFα appears to play a direct role in chronic cognitive changes and may be an independent factor in cognitive outcome after SAH (Rosi et al., 2004; Tobinick, 2009; Belarbi et al., 2012). In the aSAH population, correlation of the Hp genotype with the immune and inflammatory response has not been performed. Future studies should investigate the cytokine response and TNFα levels with the Hp 2-2 genotype. In addition, this should be performed both systemically and intrathecally in an attempt to separate an inherent immune reactivity of the Hp 2-2 vs. improved clearance of the Hp 1-1.

## BBB disruption

The blood brain barrier (BBB) is formed by brain microvascular endothelial cells (ECs) with tight junctions and astrocyte end feet encircling ECs and with the participation of pericytes. The BBB plays a fundamental role in brain homeostasis by regulating the entry of intravascular molecules from the blood into the brain (Ueno, 2007). Studies into BBB dysfunction associated with SAH are relatively sparse compared with the literature on CV (Shigeno et al., 1982; Trojanowski, 1982; Peterson and Cardoso, 1983a,b; Dóczi et al., 1986). In animals, a significant BBB permeability change has been observed at the beginning as early as 3 h after SAH, peaking at 48 h, and normalizing on day 3 (Dóczi, 1985; Dóczi et al., 1986; Germanò et al., 2007). Multiple processes may contribute to BBB breakdown after SAH, including EC necrosis/apoptosis, EC and/or pericyte contraction, and disassembly of tight junctions resulting in increased vascular permeability and formation of brain edema (Shigeno et al., 1982; Kago et al., 2006; Haorah et al., 2007; Kahles et al., 2007; Butt et al., 2011).

The EC has an active role following SAH and is critical to the BBB. Blood in the subarachnoid space rapidly stimulates the expression of cell adhesion molecules (CAMs) on the luminal surface of ECs (Gallia and Tamargo, 2006; Silva et al., 2009). This is coupled with overall reduction in protein synthesis (Foley et al., 1994), reduced expression of zona occludens 1 [ZO-1, a key cytoplasmic tight junction (TJ) accessory protein] altered expression of Claudin 5, and changes in the EC cytoskeleton, which all contribute to reduced integrity of the endothelium and BBB (Dóczi et al., 1986; Silva et al., 2009; Butt et al., 2011). CAMs and selectins expressions allow macrophages and neutrophils to bind the ECs and move in to subarachnoid space, where they phagocytose extravasated RBCs (erythrophagocytosis) and clear free Hb via CD163 mediated endocytosis of Hb:Hp complexes (Gallia and Tamargo, 2006). These trapped macrophages and neutrophils then die and degranulate in the subarachnoid space resulting in the release of intracellular endothelins and oxygen free radicals, which contribute to inflammatory-induced EC damage and BBB dysfunction (Hijdra et al., 1988; Dietrich and Dacey, 2000; Claassen et al., 2001).

In animal models, EC and BBB damage has been demonstrated to occur after systemic or intrathecal infusion of Hb (Trojanowski, 1982; Peterson and Cardoso, 1983a,b; Dóczi et al., 1986; Butt et al., 2011). Studies in models of intracerebral hemorrhage also support the finding that that exposure to cell-free Hb leads to disruption of the BBB (Xi et al., 1998; Bhasin et al., 2002; Keep et al., 2008). *In vivo* experiments suggest that Hp binding to Hb mitigates EC cytotoxicity and cell membrane modification (Schaer C. A. et al., 2013; Schaer et al., 2014). There is also limited clinical evidence that an increased Hp concentration in disease states is also protective against EC injury (Dalan et al., 2017). Since Hp 2-2 patients have lower Hp serum levels and lower Hp antioxidant properties, these patients may be predisposed to increased EC injury and BBB dysfunction after SAH (Langlois and Delanghe, 1996; Melamed-Frank et al., 2001; Dalan et al., 2017).

Accumulating evidence suggests a role for MMP-9 in the early disruption of the BBB after SAH (Sehba et al., 2004; Guo et al., 2010). MMP-9 degrades the extracellular matrix of the cerebral microvessel basal lamina, which includes collagen IV, laminin, fibronectin, and inter-endothelial tight junction proteins such as ZO-1 (Sehba et al., 2004; Suzuki et al., 2010). In cerebral ischemia and SAH, TNFα is known to be increased and may correlate with a worse clinical outcome (Dong et al., 2009; Vecchione et al., 2009; Chou et al., 2012). As mentioned, the free Hb breakdown pathway triggers the release of TNFα through the TLR4/ NFκB pathway, and TNFα is one cytokine known to increase MMP activity (Sun et al., 2010). The effect of Hp genotype on TNFα has not been studied in SAH (Lazalde et al., 2014).

No studies have been done to clearly elucidate the neuroprotective role of Hp in BBB dysfunction. But considering the fact that blood breakdown products such as Hb/heme and associated oxidative stress can contribute to BBB disruption, Hp mediated clearance of free Hb may possibly be one of the major mechanisms involved in preventing BBB dysfunction after SAH. In the context of intracerebral hemorrhage, experimentally induced hypohaptoglobinemia resulted in increased brain edema, indeed suggesting a protective role of Hp in BBB preservation (Zhao et al., 2009). One recent publication in diabetic patients suggested that Hp 2-2 patients, who have a lower plasma concentration of Hp, have increased EC apoptotic rates (Dalan et al., 2017). Future studies are also warranted to determine the effects of Hp phenotypes in EC and/or pericyte damage/function and BBB breakdown. Interestingly, the properties of Hp1-1 (86 kDa) indicate a significant molecular size advantage over Hp 2-2 (170–900 kDa) which may help it cross cell membranes and the disrupted BBB, adding another potential protective advantage (Langlois and Delanghe, 1996; Melamed-Frank et al., 2001).

## Cerebral vasospasm

Cerebral vasospasm occurs in most patients following aSAH, but only approximately 20-30% develop symptomatic cerebral vasospasm (Kassell et al., 1985). Cerebral vasospasm is related to disruption of the balance of vasodilators and vasoconstrictors (Pradilla et al., 2010). Nitric oxide (NO) is a vasodilator that is produced by ECs, neurons, and microglia and regulates cerebral vascular tone (Faraci and Brian, 1994). Following SAH, Hb released from erythrocytes can scavenge and reduce available NO, and cause delayed eNOS dysfunction (Sehba et al., 2000; Pluta, 2005, 2006; Azarov et al., 2008). Decreased levels of NO are associated with CV, and experiments increasing NO reduce cerebral vasospasm (Gabikian et al., 2002; Sun et al., 2003; Pradilla et al., 2004; Pluta et al., 2005; Pluta, 2006; Sabri et al., 2013a).

In animal models, inducing expression of Hp or therapeutic infusion of Hp is protective of the vasoactive effects from intravascular hemolysis (Boretti et al., 2009). Although, it would be intuitive to assume that Hp binding reduces NO reactivity with Hb, this does not appear to be the case (Azarov et al., 2008; Boretti et al., 2009). Rather Hp-Hb binding stabilizes the reduced Hb form and reduces NO scavenging by decreasing further free radical formation via improved Hb sequestration and augmented macrophage related clearance of Hb (Miller et al., 1997; Boretti et al., 2009; Levy et al., 2010; Cooper et al., 2013; Schaer et al., 2016).

Vasospasm is also intimately involved with inflammation and it has been shown that pro-inflammatory substances can trigger CV without blood in the CSF (Mori et al., 1994; Nagata et al., 1996; Recinos et al., 2006). CV is thought to be mediated through TLR4 activation/TNFα, and it has been shown that inhibition of TNFα prevents and resolves vasospasm in a murine model (Vecchione et al., 2009; Okada and Suzuki, 2017). In a mouse model of SAH, the administration of TLR4 antagonists intraventricularly prevented post-SAH neurological impairments and vasospasm (Kawakita et al., 2016). TLR4 knockout also suppressed vasospasm in a mouse blood injection model, with microglial TLR4 being necessary for induction of vasospasm most likely through TNFα induction (Hanafy, 2013). As stated above, timely formation of Hp-Hb complex plays a major role in preventing TLR4 activation. In a prospective study of thirty patients, those with a higher TLR4 levels on peripheral blood cells experienced increased occurrence of vasospasm and delayed cerebral ischemia (Ma et al., 2015).

Several clinical studies have shown a correlation with Hp 2-2 genotype and increased CV after aSAH (Borsody et al., 2006; Ohnishi et al., 2013; Leclerc et al., 2015). The mechanism for this benefit is not entirely clear. No study looking at Hp genotype and NO levels after aSAH has been performed. However, systemic diseases including diabetes and preeclampsia during pregnancy have indicated a correlation with Hp 2-2 genotype, lower NO levels and increased vascular tone (Sertório et al., 2013; Dahan et al., 2015). Whether Hp 2-2 has lower NO availability compared to Hp 1-1 in aSAH is a plausible, though speculative, mechanism. Similarly, whether TLR4 polymorphism can exacerbate SAH vasospasm in certain or all genotypes of Hp needs further investigation (Ferwerda et al., 2008).

## Arterial microthrombosis

It is known that SAH is associated with a hypercoagulable state, although this has been the subject of limited clinical investigation (Ikeda et al., 1997; Boluijt et al., 2015). Coagulation disorders can be divided into factors affecting (1) platelet activation, aggregation, and interaction with ECs, (2) blood coagulation, and (3) fibrinolysis (Cardenas et al., 2016). Few publications have attempted to address these factors comprehensively with contradictory results (Fujii et al., 1997; Ikeda et al., 1997; Boluijt et al., 2015). Nevertheless, studies have linked deviations in the coagulation and fibrinolytic cascade in patients with poor outcome after aSAH (Peltonen et al., 1997; Ji et al., 2014; Boluijt et al., 2015; Ramchand et al., 2016).

As might be expected in the setting of EC injury and a hypercoagulable state, arterial microthrombosis has been demonstrated in patients with aSAH in both human and *in vivo* animal model (Suzuki et al., 1990; Sehba et al., 2005; Stein et al., 2006; Friedrich et al., 2012; Sabri et al., 2012). Furthermore, the development of microthrombi is one mechanism contributing to poor outcome, and remains a target for therapeutic intervention (Suzuki et al., 1990; Stein et al., 2006; Vergouwen et al., 2008). Some authors have established that these thrombi are embolic, and on transcranial Doppler investigations, microembolic signals have been observed in up to 70% of those with SAH and this has been associated with delayed ischemia (Romano et al., 2002; Azarpazhooh et al., 2009). However, other authors have measured the frequency of microembolic signals and found their incidence to be much lower (Paschoal et al., 2015). The evidence seems to favor the development of local microthrombi in spastic, EC injured arterioles rather than systemic microemboli as the primary culprit for microthrombosis (Friedrich et al., 2012; Sabri et al., 2012).

Coagulation is inextricably linked to inflammatory cytokines known to be elevated after aSAH including TNFα (via TLR4), which activates coagulation by decreasing protein kinase C production (an antithrombotic), increasing endothelial production of tissue factor (procoagulant), and increasing platelet activation (Valone and Epstein, 1988; Kirchhofer et al., 1994; Yamamoto et al., 1999; Wagner and Burger, 2003; Pircher et al., 2012). This platelet activation is also strongly inhibited by NO, and aSAH is a depleted NO state induced by free Hb scavenging, and injury to ECs which produce NO (Sehba et al., 2000; Loscalzo, 2001; Wagner and Burger, 2003; Voetsch et al., 2004; Azarov et al., 2008; Boretti et al., 2009). Haptoglobin binding to Hb reduces inflammation and preserves NO, and this may reduce hypercoagulable complications (Figueiredo et al., 2007; Azarov et al., 2008; Boretti et al., 2009; Vecchione et al., 2009; Lazalde et al., 2014; Schaer et al., 2014, 2016; Costacou et al., 2015). Unfortunately, clinical trials have not shown a clear benefit for antiplatelet agents or systemic anticoagulation, although a clinical trial evaluating low dose systemic heparin is ongoing (NCT02501434) (Siironen et al., 2003; van den Bergh et al., 2006; Dorhout Mees et al., 2007; Simard et al., 2013). One reason for this may be our current inability to identify patients at high risk for cerebral microthrombosis.

There is also a correlation between Hp 2-2 and clinical prothrombotic sequelae, possibly secondary to increased oxidative stress associated with Hp 2-2, and reduced NO (Delanghe et al., 1997; Melamed-Frank et al., 2001; Touyz and Schiffrin, 2004; Voetsch et al., 2004; MacKellar and Vigerust, 2016). In a study of patients with spontaneous venous thromboembolism, it was found that the Hp 2-2 genotype was associated with a significantly increased risk, however this has never been studied in an aSAH population (Vormittag et al., 2005). Further evidence of hypercoagulability with Hp 2-2 is noted in cardiac literature indicating this genotype is associated with worse outcomes after cardiac bypass, and a higher rate of cardioembolic events following coronary angioplasty (Delanghe et al., 1997; Roguin et al., 2003). Clinical studies investigating spontaneous deep venous thrombosis formation in patients with aSAH show a high incidence (24%), however a correlation to Hp genotype has not been investigated (Ray et al., 2009).

Whether the Hp2-2 genotype is associated with a clinically significant hypercoagulability profile in aSAH needs further investigation, though this genotype may create a synergistic effect with the aSAH-induced hypercoagulability. The indiscriminate use of antiplatelets and anticoagulants has not proven beneficial, and determining a genetic predisposition for a hypercoagulable state would by a significant benefit for clinical trial design and therapeutic intervention.

## Scientific discrepancies

In comparison to plasma, Hp is present in the CSF in low concentrations and may be rapidly overwhelmed by the amount of degraded erythrocytes in the CSF (Galea et al., 2012). There are still arguments within the community whether Hp and CD163 would be significantly present within the CNS to be able to deal with the supra-physiological levels of hemoglobin and met-hemoglobin. Systemic Hp expression is upregulated as an acute phase reactant following aSAH, though this increase does not clearly translate to the CSF (Galea et al., 2012). Nevertheless, it is known that oligodendrocytes are capable of producing some Hp, and increased expression has the potential to serve as a therapeutic mechanism to reduce TLR4 activation and subsequent inflammation (Zhao et al., 2009). It also appears that Nuclear factor related factor 2 (Nrf2) activator in the context of ICH could robustly increase production of Hb not only in the blood but also in the brain, providing an intriguing solution for modulating Hp levels in the brain (Zhao et al., 2007, 2009).

There has also been interest in the role of CD163 as a mediator for varying effects of Hp-Hb clearance. There does seem to be a higher affinity of Hp2-2 bound Hb for CD163; however, the internalization of the bound complex (Hp-Hb-CD163) may be higher for Hp1-1, although this is uncertain and requires further investigation (Kristiansen et al., 2001; Asleh et al., 2003; Na et al., 2005; Lipiski et al., 2013).

Most work on Hp genotype has focused on peripheral markers and not CSF. This remains a particular problem to aSAH since it is thought that the ictus and extravasation of blood into the CSF rapidly overwhelms the Hp binding capacity, regardless of phenotype (Galea et al., 2012). So why would Hp 1-1 phenotype be protective when Hp primarily exists in the circulation outside of the BBB? One explanation is that the size of Hp 1-1 is significantly smaller than Hp 2-2, and it may cross the BBB into the CSF more easily, thus improving the hypo-Hp state (Langlois and Delanghe, 1996). However, this runs counter to data which shows a lower concentration of CSF Hp in patients with Hp 1-1, and this is suspected to be due to improved macrophage uptake (Galea et al., 2012).

Investigation into the temporal Hp concentration in the CSF after aSAH remains necessary to understand the utilization of Hp over time, clearance of Hp-Hb, and potentially movement of peripheral Hp into the CSF (and vice versa, Hb into the periphery). More reliable studies investigating bound CSF Hp (i.e., Hb-Hp) vs. free CSF Hp will help elucidate the mechanism of blood clearance and Hp protection after SAH.

The glymphatic pathway (or meningeal lymphatic system) for clearance of CSF solutes has become an interesting topic of investigation. There is evidence that increasing solute size limits glymphatic/lymphatic system clearance, and furthermore, aSAH reduces its function (Iliff et al., 2012; Gaberel et al., 2014; Bacyinski et al., 2017). Because Hp1-1 is smaller than Hp2-2, it is possible that Hp1-1 bound hemoglobin is cleared more readily or does not obstruct clearance of other brain metabolites, though this requires further investigation.

Lastly, several limitations exist for experimental studies of Hp in mice. First, mice only have a single Hp genotype which corresponds to human Hp1, and therefore mice only express the Hp1-1 phenotype (Levy et al., 2006). Second, preclinical studies administering Hp utilize mixed haptoglobin solutions, consisting of multiple Hp phenotypes (Graw et al., 2016, 2017; Shi et al., 2016). These limitations make interpretation of Hp's role in brain hemorrhage pathophysiology difficult to assess. Future studies investigating Hp in aSAH and other cerebral hemorrhages should utilize Hp2 mutant mice in addition to the Hp1 expressing wild-type mice.

## Conclusions and future directions

Lysis of RBCs and release of free Hb/heme drives the majority of the pathological process involved in delayed brain injury following SAH. Haptoglobin and its polymorphism has been extensively studied in other disease states, so much so that a human plasma-derived Hp was developed and is being utilized in the treatment of hemolysis (Schaer D. J. et al., 2013). Evidence from both preclinical and clinical studies support the notion that polymorphism in haptoglobin is a culprit in the delayed brain injury phase of SAH. Future efforts should focus on larger scale studies dealing with Hp polymorphism, on deciphering the molecular mechanisms by which Hp function and possibly testing Hp infusion as therapy for SAH patients suffering form delayed brain injury.

## Author contributions

The conception of this article was by SB and JG. Both the writing and editing was performed by all authors. Authors SB, PP, DM, HZ contributed to the initial manuscript by writing individual portions of the manuscript. These were combined and edited by SB, and then submitted to HC, JG, PD, JC, and SD for expert editing and final preparation. Both JL and JA reviewed and critically edited the content of the manuscript.

### Conflict of interest statement

The authors declare that the research was conducted in the absence of any commercial or financial relationships that could be construed as a potential conflict of interest.
